# Bariatric surgery: the challenges with candidate selection, individualizing treatment and clinical outcomes

**DOI:** 10.1186/1741-7015-11-8

**Published:** 2013-01-10

**Authors:** KJ Neff, T Olbers, CW le Roux

**Affiliations:** 1 Experimental Pathology, UCD Conway Institute, School of Medicine and Medical Sciences, University College Dublin, Belfield, Dublin 4, Dublin, Ireland; 2 Department of Bariatric Surgery, Carlanderska Hospital, Gothenburg, Sweden; 3 Department of Gastrosurgical Research and Education, University of Gothenburg, 5-413 45 Gothenburg, Sweden

**Keywords:** Bariatric surgery, obesity, pre-operative assessment, postoperative outcomes.

## Abstract

Obesity is recognized as a global health crisis. Bariatric surgery offers a treatment that can reduce weight, induce remission of obesity-related diseases, and improve the quality of life. In this article, we outline the different options in bariatric surgery and summarize the recommendations for selecting and assessing potential candidates before proceeding to surgery. We present current data on post-surgical outcomes and evaluate the psychosocial and economic effects of bariatric surgery. Finally, we evaluate the complication rates and present recommendations for post-operative care.

## Background

The rates of obesity are increasing with at least 300 million people worldwide now classified as obese [1]. Obesity is associated with reduced life expectancy, increased morbidity and mortality, and greater healthcare costs [2,3]. Bariatric surgery is more effective than non-surgical treatments of obesity with a reduction in overall mortality of 30% demonstrated in surgical recipients [4-7]. Greater reductions are seen in cancer and diabetes mortality [6,7].

Recommendations for referring candidates for bariatric surgery are available but they seldom give guidance to help a specific patient. We will examine the evidence for the magnitude and duration of the positive effects and negative side effects of bariatric surgery. Finally, we will offer a synopsis of the data on post-operative outcomes, and on what to expect in the months and years following surgery.

## Review

### Individualizing treatment

#### Available options in bariatric surgery

Several bariatric procedures are available. The most commonly performed procedures are Roux-en-Y gastric bypass (RYGB), adjustable gastric banding (AGB), and sleeve gastrectomy (SG) [8]. Biliopancreatic diversion, with or without duodenal switch (BPD and BPD-DS), is less commonly performed but is often considered in extremely obese individuals [9]. All procedures can be performed laparoscopically with a lower rate of complications such as wound infection and incisional hernias [10].

**Figure 1:**
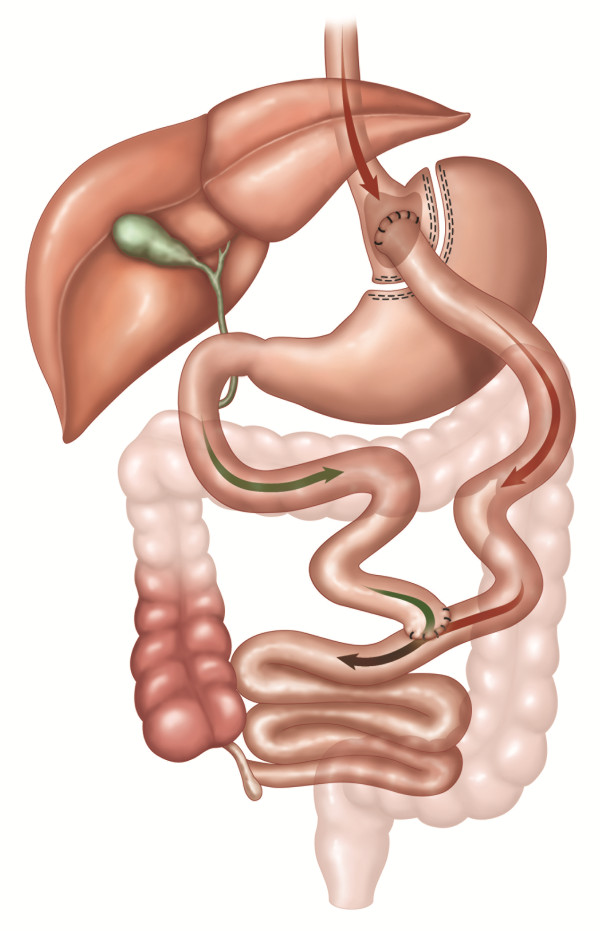
**RYGB: Roux-en-Y gastric bypass**. An upper gastric pouch, of 15 to 30 mL in volume, and a lower gastric remnant is formed from the stomach. The jejunum is divided some 30 to 75 cm distal to the ligament of Treitz, and anastomosed to the gastric pouch. The distal jejunum is brought up as a 'Roux-limb'. The excluded biliary limb, including the gastric remnant, is anastomosed to the bowel some 75 to 150 cm distal to the gastrojejunostomy. The included figures are the property of Johnson and Johnson and Ethicon Endo-Surgery (Europe). They are reproduced here with their kind permission.

In RYGB, the stomach is divided into an upper gastric pouch, which is 15 to 30 mL in volume and a lower gastric remnant. The gastric pouch is anastomosed to the jejunum after it has been divided some 30 to 75 cm distal to the ligament of Treitz; this distal part is brought up as a 'Roux-limb'. The excluded biliary limb, including the gastric remnant, is connected to the bowel some 75 to 150 cm distal to the gastrojejunostomy (see Figure 1).

**Figure 2:**
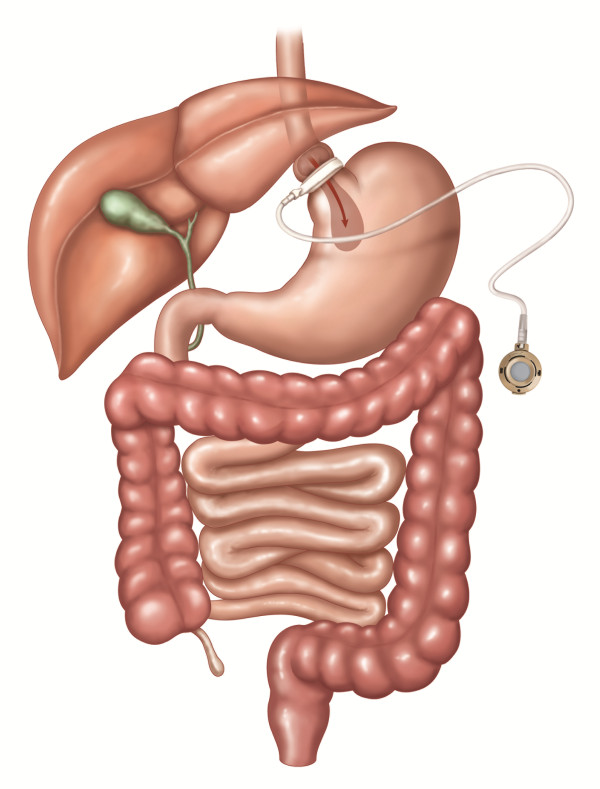
**AGB: Adjustable gastric band**. A band with an inner inflatable silastic balloon is placed around the proximal stomach just below the gastroesophageal junction. The band is adjusted through a subcutaneous access port by the injection or withdrawal of solution. The included figures are the property of Johnson and Johnson and Ethicon Endo-Surgery (Europe). They are reproduced here with their kind permission.

In AGB, a band with an inner inflatable silastic balloon is placed around the proximal stomach just below the gastroesophageal junction. The band can be tightened through a subcutaneous access port by the injection or withdrawal of a saline solution [11] (see Figure 2).

**Figure 3:**
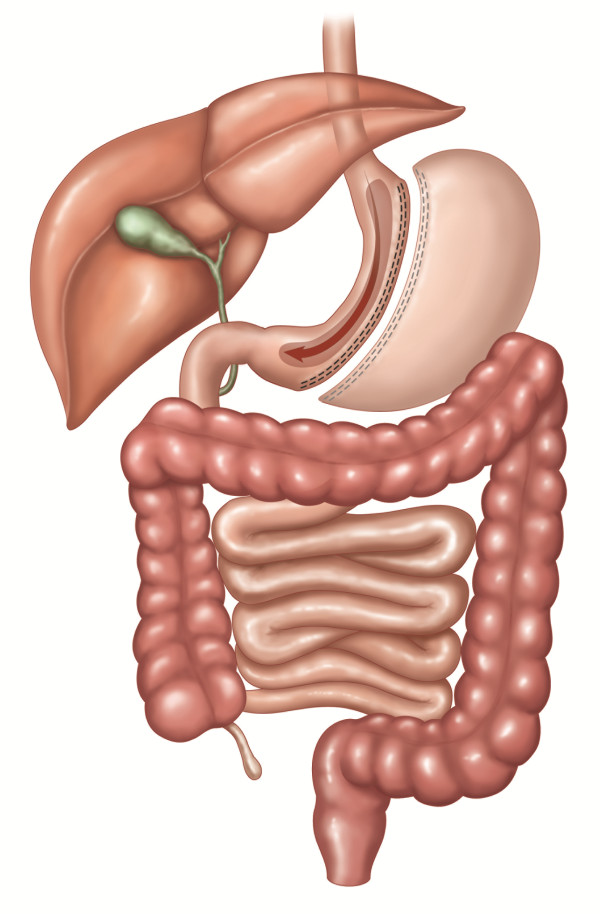
**SG: Sleeve gastrectomy**. The stomach is transected vertically creating a gastric tube and leaving a pouch of 100 to 200 mL. The included figures are the property of Johnson and Johnson and Ethicon Endo-Surgery (Europe). They are reproduced here with their kind permission.

In SG, the stomach is transected vertically over a 34 or 36F bougie creating a gastric tube and leaving a pouch of 100 to 200 mL (see Figure 3). Although many regard SG as a restrictive procedure, it is increasingly recognized as a metabolic procedure [12].

**Figure 4:**
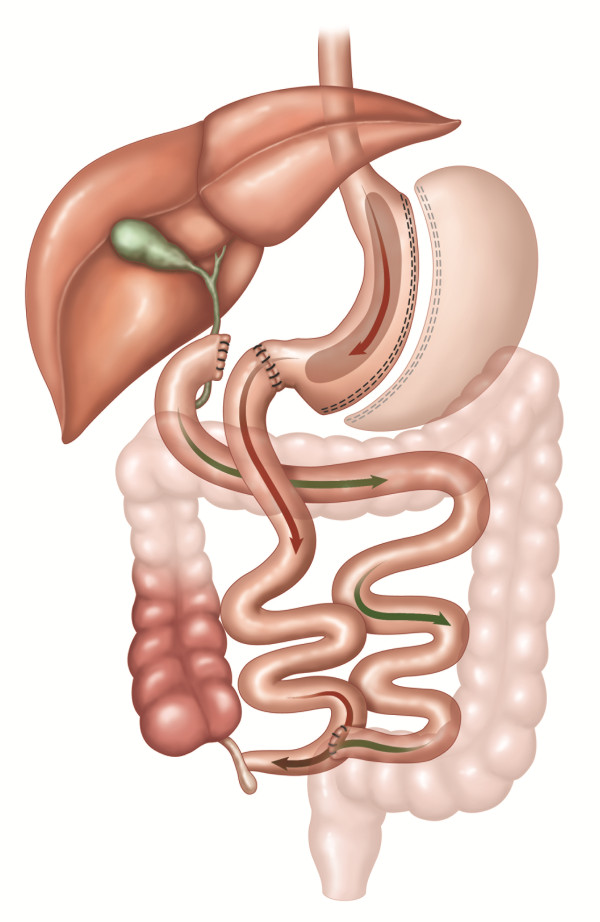
**BPD: Biliopancreatic diversion**. A 400 mL gastric pouch is formed from the stomach. The small bowel is divided 250 cm proximal to the ileocecal valve and is connected to the gastric pouch to create a Roux-en-Y gastroenterostomy. An anastomosis is performed between the excluded biliopancreatic limb and the alimentary limb 50 cm proximal to the ileocecal valve. In BPD-DS, a vertical sleeve gastrectomy is constructed and the division of the duodenum is performed immediately beyond the pylorus. The alimentary limb is connected to the duodenum, whereas the iliopancreatic limb is anastomosed to the ileum 75 cm proximal to the ileocecal valve. The included figures are the property of Johnson and Johnson and Ethicon Endo-Surgery (Europe). They are reproduced here with their kind permission.

BPD involves a partial gastrectomy that results in a 400 mL gastric pouch [13]. The small bowel is divided 250 cm proximal to the ileocecal valve, and the alimentary limb is connected to the gastric pouch to create a Roux-en-Y gastroenterostomy. An anastomosis is performed between the excluded biliopancreatic limb and the alimentary limb 50 cm proximal to the ileocecal valve (see Figure 4). In BPD-DS, a vertical SG is constructed and the division of the duodenum is performed immediately beyond the pylorus. The alimentary limb is connected to the duodenum, whereas the iliopancreatic limb is anastomosed to the ileum 75 cm proximal to the ileocecal valve [14].

Endoscopically placed synthetic duodenojejunal bypass liners such as the EndoBarrier^® ^have been recently developed and are associated with a mean weight loss of 10% to 20% [15,16]. These devices establish duodenal exclusion and result in greater weight loss than diet and exercise alone up to 12 weeks post-insertion [17]. They may also improve glycemic control in those with type 2 diabetes mellitus (T2DM) [18].

However, long-term data remain to be reported and the device is often poorly tolerated [18]. Complications include sleeve migration and obstruction, which can occur with a frequency of 15% to 20% [15,16,18]. It can also be difficult to insert the device with placement failure in up to 13% [18]. While the concept of endoscopic techniques such as Endobarrier^® ^remains attractive, the permanence of the weight loss and the clinical role of the device itself remain to be determined.

Other techniques for the treatment of obesity include the intra-gastric balloon, which can be effective for short-term weight loss [16]. However, these newer techniques remain in the experimental realm and data on long-term clinical efficacy are not available. The EndoBarrier^® ^may not be any better or worse than gastric balloons or very low calorie diets at reducing operative risk in patients with extreme obesity. The device may be developed for use in those with diabetes and obesity who decline laparoscopic bariatric surgery. If other non-surgical treatments such as exogenous satiety gut hormones, or weight loss maintenance diets, can show that the weight loss after removal of the EndoBarrier^® ^can be maintained, then a comparison with established bariatric procedures may be feasible.

### Candidate selection and pre-operative assessment

Patient selection criteria for bariatric surgery include body mass index (BMI), the presence of co-morbidities and a history of prior weight loss attempts. National Institute of Clinical Excellence (NICE) and National Institutes of Health (NIH) guidelines state that bariatric surgery should be offered to patients with a BMI of 35 to 40 kg/m^2 ^who have obesity related conditions such as diabetes mellitus or obstructive sleep apnea, or in those with a BMI of 40 kg/m^2 ^or greater regardless of weight related co-morbidities [19,20]. Bariatric surgery for individuals with a BMI less than 35 kg/m^2 ^with obesity related co-morbidities is under investigation but is not currently recommended [21].

If a candidate meets the criteria for surgery, then a multi-disciplinary team assessment is made as to the suitability of the candidate. This is a complex process involving psychological, surgical, dietetic and medical review. The individual must be physically and psychologically fit to proceed to surgery. Expectations must be managed and a determination must be made as to the individuals' ability to comply with post-operative care. The decision to operate will take into account the benefits the candidate is likely to gain, and the risks peri-operatively and post-operatively.

This is an individualized assessment, and the role of the psychologist and/or psychiatrist should be central. Some reports suggest an increased risk of suicide after bariatric surgery, although the etiology remains unclear [6]. Major failures of bariatric surgery are due to poor psychological adaptation, especially if the patient's expectations were not adequately managed. All candidates should be given the correct and realistic information on what the procedure can achieve. If this is addressed, then the risk of surgical failure can often be mitigated [22,23]. This personalized assessment is a vital part of the pre-operative assessment.

For each patient the benefits of the procedure should outweigh the operative risk. In general, obese patients have an increased prevalence of cardiopulmonary disease that may be undiagnosed pre-operatively [24]. An individualized pre-operative assessment should be completed by a multi-disciplinary team [25]. Pre-operative investigations should focus on screening for cardiac arrhythmia, prolonged QT syndrome, and cardiomyopathy [25]. Almost 70% of individuals awaiting bariatric surgery can be diagnosed with obstructive sleep apnea, with over 40% meeting the criteria for severe disease [26,27]. However, this is not associated with a greater rate of peri-operative complications [28]. Male gender, age older than 50 years, congestive heart failure, peripheral vascular disease and renal impairment are associated with greater mortality [29].

### Predicting outcomes

Once the candidate has been selected, then the appropriate procedure must be chosen. Unfortunately, there is no evidence based medical approach for procedure selection, and this remains one of the most frustrating shortfalls in bariatric practice for clinicians and patients. Clinicians take a pragmatic approach to the choice of procedure, and the decision is determined by the individuals' clinical phenotype, the aims of therapy, and peri-operative risk.

Clinicians and patients can often be disappointed when surgical outcomes are not as impressive as may have been hoped and look for objective evidence that can allow them to predict outcomes. The most damaging outcome of surgical procedures that do not perform as expected is to blame the recipient for not performing well enough with regard to diet and exercise, when in fact, the failure is almost always rooted in biology. Weight loss prediction is one of the common aims of these predictive models. Pre-operative weight loss can predict post-operative weight loss, putatively as a marker of 'intrinsic motivation' [30,31]. These data are contentious and not without bias as many of them are retrospective uncontrolled studies [32]. The available prospective data are of short duration [31,32]. A systematic review shows that 50% of the published results find that pre-operative weight loss has an association with weight change post-operatively, and the remaining 50% refute these findings [32]. In the context of this evidence, pre-operative weight loss cannot be relied on to predict surgical outcomes at this time.

Genetic disorders such as melanocortin-4 receptor (MC4R) deficiency may have an inherent physiological role in these mixed results, as animals with MC4R deficiency have a resistance to weight loss after bariatric surgery [33]. There is interest in these genotypes, as identifying them may aid the prediction of outcomes. However, identified phenotypes such as MC4R are not common in the obese population, with heterogeneous mutations identified in less than 3% of European and North American obese cohorts [34]. Culprit genes that have been associated with obesity, with approximately 20 implicated to date, are still only found in 5% of obese people [35].

Investigation of genetic factors that may predict individual responses to bariatric surgery is ongoing and controversial [35-37]. While certain genotypes are associated with improved outcomes after bariatric surgery, they are not procedure specific and, therefore, while potentially aiding prediction of weight loss post-operatively, will not aid procedure selection [35-37]. It is also well recognized that the genetic influence on obesity may be much more complex than we currently understand, with the inevitable influence of environment making the situation less clear. For now, study of identified genotypes, with a correlation between genotype and treatment outcomes, may answer questions on the clinical utility and predictive ability of genotyping in bariatric surgery [33].

To date, any potential genetic markers or biomarkers of weight loss following bariatric surgery have been limited by clinical utility, and sensitivity and specificity [38,39]. Some data identifying potential markers of weight loss are inadequately controlled and unmatched [38]. The positive findings are in the context of complex interactions, without clinically usable tests that could be applied in daily practice [39].

While there is some potential in this field, usable techniques are still many years away [40].

The factors most consistently negatively associated with post-operative weight loss include higher BMI levels and personality disorders [32]. Given the impact of psychological markers on outcomes, techniques such the artificial neural network, which can incorporate psychological and biological measurements, have been tested to predict surgical outcomes [41]. Such techniques rely on established data that have been shown to effect outcomes, but may incorrectly predict response in as many as 30% of bariatric surgery recipients [41]. These models are multi-factorial, prone to bias and socio-cultural differences [41]. They are also time-consuming and expensive [41]. As the techniques are refined, we may develop a useful model that could be employed in routine practice, although we have not arrived at that point as of yet.

While weight loss remains difficult to predict, there are increasing amounts of data on prediction of diabetes remission. Markers of insulin secretion, such as C-peptide may aid pre-operative prediction of diabetes remission [42]. These results report an increased rate of diabetes remission with higher C-peptide levels [42,43]. The highest cut-off can predict diabetes remission with a specificity of approximately 90% [42]. Shorter duration of diabetes, lower glycosylated hemoglobin (HbA1c) levels and insulin independence are also associated with a higher post-operative remission rate [43,44]. These data illustrate a role for C-peptide to be used in conjunction with clinical data to predict diabetes remission. If a validated, sensitive and specific model were developed then it may aid procedure selection. However, the models currently studied can provide great specificity, but only at the cost of sensitivity [45].

The use of incretin and bile acid profiles has been investigated for use in predicting weight loss and metabolic outcomes following bariatric surgery [46,47]. The findings suggest that the restoration of peptide YY (PYY) and glucagon-like-peptide- 1 (GLP-1) secretion following RYGB contribute to satiety and weight loss [46,48]. Bile acids also have a role in this process, and the mechanisms underlying this are currently being elucidated [47]. Increased bile acid delivery to the terminal ileum can improve satiety and enhance weight loss [47]. However, these changes occur after surgery, and there is no current evidence that would allow them to be used to select candidates pre-procedure.

Similarly, while the restoration of the PYY and GLP-1 response is associated with satiety and weight loss in RYGB, as opposed to AGB, there are no strong data on differences within this group that allow us to predict the degree of weight loss following RYGB based on the incretin or bile acid response [48,49]. There are data demonstrating a progressive rise in PYY and GLP-1 following RYGB that is associated with increased satiety but without noted differences within the group [49]. The relationship between absolute incretin or bile acid levels, or trends, and weight loss remains to be determined. At this time, the restoration of incretin secretion and increased serum levels of bile acid are associated with enhanced satiety and weight loss, but they cannot be used to predict weight loss [46-49].

### Procedure selection

For now, procedure selection is best informed by the candidates' objectives and by how they want to live their lives after surgery. As a primary aim of surgery, the efficacy of weight loss associated with each procedure must be considered. RYGB results in greater weight loss than AGB, although good quality post-operative care can improve the weight loss following AGB, with results comparable to RYGB [50-52]. AGB is associated with a lower rate of immediate post-operative complications but also a higher rate of re-operation for insufficient weight loss [50-52]. The associated mortality rate is higher with RYGB than with AGB but still less than 0.3% [50,51].

Weight loss is comparable between RYGB and SG in the short term [53,54]. Some studies suggest that more patients will regain weight in the medium-term after SG [55]. BPD/BPD-DS results in greater weight loss, but with higher complication rates, than RYGB [56,57]. Therefore, the greatest weight loss would likely be achieved with BPD/BPD-DS. However, this is not generally agreed. BPD/BPD-DS may not be suitable for high-risk operative candidates and some randomized controlled trials have shown no additional benefit of the extra weight loss above RYGB [58].

There are variances in outcome between national health systems, with AGB considered a superior bariatric procedure in systems where there is an excellent post-operative care pathway [52]. This implies that AGB can have comparable results providing that the post-operative care is planned and provided by experienced clinicians.

With regard to diabetes remission or treatment, RYGB offers a greater rate of remission than AGB [50,51]. SG has a remission rate comparable to RYGB in the short-term, but a higher rate of relapse in the medium-term [59]. BPD and BPD-DS may offer a higher rate of diabetes remission than RYGB or AGB [60-62]. While AGB is the least effective in inducing diabetes remission, it can offer substantial improvements in diabetes control, which are greater than those offered by medical therapy in obese cohorts [63,64].

Other conditions can influence a decision on bariatric surgery. Respiratory disease may improve more significantly with greater weight loss [65]. Therefore, those with obstructive sleep apnea (OSA) or asthma could theoretically be considered for more consistently efficacious procedures such as BPD/BPD-DS or RYGB. Conversely, SG and AGB are associated with deteriorations in gastro-esophageal reflux disease (GERD) and, therefore, should be avoided in this cohort [55,66]. In GERD, RYGB is increasingly considered as the treatment of choice as it can remediate the GERD due to the reduction in the stomach pouch and prevention of esophageal reflux [67].

In summary, candidate selection and preparation is key to achieving good surgical outcomes. Each procedure should be considered for each individual, and the data to date do not support the application of a generic selection based on body weight, diabetes or other co-morbidities. The choice of procedure is a complex process with the patient and their interests at its core. The surgeon's experience to deal with the inevitable complications of each procedure and to manage long-term follow-up care remain dominant considerations.

In those with diabetes, BPD/BPD-DS offers the highest rate of remission, but also the highest complication rates. RYGB and SG are comparably efficacious in treating diabetes in the short-term, but questions remain regarding the medium to long-term. It should be noted that the volume of data for RYGB is greater than that for SG and BPD/BPD-DS. For those with GERD, RYGB should be the treatment of choice. SG and AGB should be avoided.

AGB can also lead to weight loss and diabetes remission and can offer greater control than medical therapy, even if remission is not achieved. It should be noted that the choice of AGB should take into account the availability of good quality post-operative care. AGB may be suitable for those who wish to lose weight and improve diabetes control, but not remission, and are at higher surgical risk.

### Clinical outcomes

The outcomes following bariatric surgery vary between procedures, and predicting individual outcomes following bariatric surgery is difficult, for the reasons outlined in the previous sub-sections. We will review each outcome individually and compare between modalities below.

#### A. Airway: Obstructive sleep apnea (OSA) and asthma

Bariatric surgery is associated with impressive remission rates for OSA (68). However, bariatric surgery can improve the severity of OSA more frequently than resulting in full remission, and the improvement can still leave the individual in a moderate or severe category [68]. The symptoms of asthma can improve after bariatric surgery but the mechanism of this effect is unknown, although reduction of subcutaneous tissue with improvement of the restrictive effect on the chest wall may be involved [69].

#### B. Body weight

Bariatric surgery effectively induces weight loss, but the degree varies between procedures [8-10]. RYGB results in greater weight loss than AGB in most studies [51]. The quality of the post-operative care affects weight loss after AGB, with good quality post-operative care resulting in comparable weight loss to RYGB [52]. Weight loss is comparable between RYGB and SG at 36 months post-operatively but long-term data are pending [53]. Weight regain can be frequent following SG [55]. BPD results in greater weight loss, but higher complication rates, than RYGB, with comparable metabolic and quality of life outcomes [56,58].

Weight loss usually reaches a maximum twelve months post-operatively, and some weight regain is common thereafter. The mean ten-year weight reduction is 25% for RYGB and 13% for AGB [4].

#### C. Cardiovascular and cardiac disease

Obesity is a risk factor for cardiovascular disease [70]. The available data over a median of almost twenty years show that bariatric surgery is associated with reduced cardiovascular mortality and morbidity [71]. In RYGB a reduction of cardiovascular morbidity of more than 50% is seen when compared to BMI and age matched controls [6,71]. The mechanism is unclear but improvements in glucose metabolism, blood lipid profiles and hypertension probably contribute [71,72]. The reduction in hypertension and dyslipidemia does remit somewhat post-operatively but both remain reduced from baseline at ten years [6,7].

Cardiomyopathy in obesity is associated with left ventricular hypertrophy and diastolic dysfunction with a longer exposure to obesity associated with worse cardiac function and larger ventricular mass [73]. Left atrial dilatation and systolic dysfunction can also develop [74]. This is likely due to a combination of increased cardiac output and increased circulatory volume [74]. Bariatric surgery has been shown to result in improved cardiac function and 'reverse remodeling' of the left ventricle up to three years post-operatively [75].

#### D. Diabetes

There is a strong association between obesity and diabetes with approximately half of those diagnosed with T2DM classified as obese [76]. Bariatric surgery can induce remission of diabetes by inducing weight loss [61]. There are also enteroendocrine effects following RYGB and BPD, achieving greater remission rates for diabetes when compared to patients who have had similar weight loss after AGB [61]. Diabetes remission is greatest for patients undergoing BPD-DS, followed by RYGB and then AGB [61]. SG has a comparable remission rate to RYGB [59,77]. The remission of diabetes following bariatric surgery can be transient, with 72% free of diabetes two years after bariatric surgery but only 36% remaining free of diabetes at ten years [4].

The most reliable long-term prospective data comes from the Swedish Obese Study in which the majority of participants underwent vertical banded gastroplasty with the remaining undergoing RYGB or AGB [4]. Subgroup analyses report that the RYGB group (N = 34) had a greater reduction in serum glucose at ten years than the rest of the cohort (N = 608) [4]. However, rates of diabetes according to subgroup and other prospective data for RYGB specifically are not available. Retrospective data at nine years following RYGB show a reduced rate of medical treatment of diabetes by more than 65% in parallel with a reduction in mortality of more than 70% [78]. The improvement in mortality was primarily due to a decrease in the number of cardiovascular deaths.

Shorter duration of diabetes, lower HbA1c levels and insulin independence are associated with a higher post-operative remission rate [44].

The presence of diabetes could influence the choice of bariatric procedure with RYGB, BPD and SG considered to result in remission of diabetes in a significant proportion of candidates. In those who do not achieve remission, bariatric surgery, including AGB, results in better glycemic control and a reduced medication burden compared to medical treatment [63,64]. Emerging data also suggest that bariatric surgery may facilitate remission of microvascular complications, such as microalbuminuria [79].

#### E. Economic

Obese individuals are more than twice as likely to take sick leave and almost three times as likely to avail of disability benefits [4]. Medical costs are significantly higher for obese individuals, mainly due to the cost of diabetic, hypertensive and lipid therapy, but with additional costs secondary to analgesia, respiratory and psychiatric treatments [80,81]. When classified by BMI, patients in the highest ranges spend more on healthcare [81].

While the presence of obesity may be secondary to lower socio-economic status rather than causative, bariatric surgery has been shown to result in increased productivity and reduced sick leave [82,83]. It is more costly than non-surgical management of obesity in the short-term but a return of investment can be achieved within four years [84].

Medication prescription is reduced by bariatric surgery with resultant reductions in healthcare costs that can persist for up to 20 years [84,85]. Cost effectiveness may also be achieved through reduced healthcare system utility due to the reduction in obesity related co-morbidities [85]. However, the modeling used in this type of cost assessment is open to criticism and there is a dearth of controlled prospective data.

#### F. Functional

Basic activities of daily living such as walking and personal hygiene can be affected by severe obesity, and this loss of autonomy can be extremely distressing for the affected individuals [86]. Joint pain, including lower back pain, is common in obese populations and can impinge on individual functional status [87]. Bariatric surgery results in improved function status, reduced levels of back pain and greater levels of independence [87].

#### G. Gonadal function and fertility

In men, obesity can result in erectile dysfunction, reduced serum testosterone levels and reduced sperm quality [88,89]. Bariatric surgery is associated with increased serum testosterone levels but may paradoxically result in a deterioration in sperm quality [90,91]. There are no controlled prospective data to evaluate the effect of bariatric surgery on male fertility.

In women, obesity is associated with high rates of ovulatory dysfunction, increased risk of spontaneous abortion and increased materno-fetal risk in pregnancy [92]. There is evidence that weight reduction via bariatric surgery can improve ovulatory cycles and reduce hyperandrogenism in women [93]. It also probably reduces materno-fetal risk, although the current evidence is mainly limited to observational data [94,95]. To date, there are no randomized controlled data or long-term prospective data available and, therefore, no strong recommendation can be made on advising reproductively active women considering bariatric surgery.

#### H. Perceived health status

People who are classified as obese often report poor health perceptions and altered mood [96]. Psychiatric co-morbidities including anxiety and depression are also common [97]. Bariatric surgery improves quality of life and perceived health status, with changes seen in the first year and benefit retained up to ten years [96]. Depression, aggression and low self-concept can all be improved by bariatric surgery [97]. The improvements in perceived health status and quality of life may be correlated with weight loss, with superior results following RYGB compared to AGB [98]. However, factors other than weight loss may be responsible for these psychological benefits as the improvements have been reported in the immediate post-surgical phase [99].

#### I. Image

Body image dysphoria is found in high frequency in obese cohorts but this sometimes improves post-operatively [86,100]. The improvement in body image satisfaction is associated with improved quality of life scores, and the improvements continue for at least two years following surgery [101,102]. Weight regain is associated with deterioration in self-concept and body image and can be associated with depressive symptoms [102]. In general, changes in body image are very unpredictable.

Eating disorders are common in obese populations [101]. Some evidence suggests that bariatric surgery can be associated with remission of eating disorders, particularly binge eating disorder [103]. The persistence of these disorders is associated with poor outcomes, and eating behavior needs to be regularly reviewed post-operatively [103].

#### J. Junction of gastro-esophagus

The presence of obesity and GERD has been linked with pre-malignant metaplasia of the gastro-esophageal junction, and frank adenocarcinoma of the esophagus [104]. RYGB can reduce the symptoms of GERD and is associated with regression of pre- malignant metaplasia [67,105]. There is concern that AGB may worsen symptoms in a significant proportion of recipients [66]. SG has also been associated with worsening GERD, and patients with pre-existing disease may not be suitable candidates [55].

Surgical treatment of GERD can be ineffective in obese populations, and RYGB can be considered before fundoplication in this group given the improved outcomes [67]. Therefore, the presence of GERD supports use of RYGB as a first line procedure.

#### K. Kidney function

While the measurement of glomerular filtration rate in obese cohorts is not well validated, obesity is noted to result in higher rates of chronic kidney disease (CKD) independent of the effect of co-morbid diabetes mellitus, hypertension or dyslipidemia [106,107]. Renal parameters such as serum creatinine and urinary protein excretion can improve after bariatric surgery, but at present it is unknown if the potential benefits outweigh the risks in those with CKD, given the greater peri-operative risk associated with renal impairment [29,108,109].

#### L. Liver

Liver disease such as hepatosteatosis, non-alcoholic steatohepatitis (NASH), hepatic fibrosis, and cirrhosis are all associated with obesity [110]. Bariatric surgery improves the histological appearance of the liver and can lead to regression of established liver disease [111]. However, these data are often uncontrolled and some authors have reported worsening in fibrosis rates after bariatric surgery [111]. The presence of fibrotic liver disease needs to be considered in the decision to proceed with surgery and in follow-up plans post-operatively.

#### M. Medication

Bariatric surgery results in a significant cost reduction in glycemic, lipid and antihypertensive therapy that can take effect within two weeks of surgery [84,85]. There are additional therapies needed following bariatric surgery, with increased prescription of GERD therapy with some procedures [80]. The need for increased GERD treatment and ongoing mineral and vitamin supplementation can partially offset the cost reductions in diabetic and cardiovascular medication [80,85].

#### O. Other

There is emerging evidence that weight loss using bariatric surgery may reduce the incidence of cancer [112]. It seems that the protective effect is strongest for women, and the reduction of risk may be as high as 60% [6,112,113]. The mechanisms underlying this apparent risk reduction are unclear, but may involve mediation of inflammatory pathways and attenuation of obesity associated hyperinsulinism [112].

### Morbidity and mortality after bariatric surgery

Surgical complications can be defined as early or late, depending on if they occur within the first thirty post-operative days or afterwards. There is a wide range in the reported complication rates. The benchmark for bariatric centers of excellence is the Longitudinal Assessment of Bariatric Surgery consortium [114]. Mortality rates after bariatric surgery are low with a mortality rate after RYGB of 0.3% [114]. Pulmonary and venous thrombo-embolism are early complications and occur in less than 0.5% of bariatric surgery recipients [115]. Other complications can be specific to the modality and include the following.

#### 1. Anastomotic leak and bowel perforation

Anastomotic leak is a feared early complication. Higher BMI, male gender, re-operation, older age and surgeon's experience are all associated with higher rates of anastomotic leakage [115,116]. Leakage can occur at any of the anastomotic junctions in RYGB, SG or BPD/BPD-DS and can result in severe peritonitis, sepsis, and multi-organ failure. Enteric leaks require emergent operative treatment in the context of hemodynamic instability or peritonitis.

Anastomotic leakage appears most commonly at the gastrojejunostomy in RYGB and the incidence associated with mortality is 0.1% [116,117]. The incidence of leakage is up to 3.6% in SG and most commonly occurs as a defect in the staple line [118]. In BPD-DS, leakage from the staple line is more common than anastomotic leakage and the total enteric leakage rate is 5% [119].

In AGB, there is no anastomosis and gastro-esophageal perforation is an uncommon early complication that can result in peritonitis and abdominal sepsis with an incidence of less than 0.5% [120].

#### 2. Hemorrhage

Hemorrhage is an early complication that occurs in up to 4% of patients [121]. Using finer anastomotic closure techniques can reduce bleeding rates [122]. The presence of diabetes mellitus has been associated with a higher risk of post-operative hemorrhage [123]. In SG, hemorrhage has an incidence of up to 5.6%, but there is a large range in reported data that is likely explained by surgical experience, the complexity of the case intra-operatively, and the use of buttress material [124,125].

#### 3. Bowel obstruction

Internal hernias can cause bowel obstruction, and can occur early or late post-operatively. The reported frequency ranges from 0.4% to 5.5% in RYGB [126,127]. Long-term data over seven years record a hernia rate of 38% in BPD/BPD-DS [128]. A laparoscopic approach may result in higher rates, but new surgical techniques where the mesentery windows are surgically closed may reduce the rate to as low as 1% [129].

#### 4. Anastomotic stricture

Anastomotic stricture is a late complication that can occur at any of the anastomotic sites. It is commonly described at the gastrojejunostomy in RYGB and is associated with dysphagia and vomiting [130]. The mean incidence of gastrojejunal stricture is approximately 10%, but rates as high as 20% are reported [130]. The laparoscopic approach and use of circular staplers to make the gastrojejunal anastomosis may result in higher rates of stricture [131].

#### 5. Other complications

Incisional hernias can occur but are less common with the increased use of laparoscopic techniques [10]. Marginal ulcers are usually late complications of bariatric surgery and occur in 2% of patients within the first post-operative year, and then in 0.5% for up to five years [132]. Proton pump inhibition is the preferred treatment but ulcers can be refractory and may require revisional surgery [133].

#### 6. Complications specific to AGB

Band migration is becoming less common since the introduction of the 'pars flaccida' technique and individually sized bands, with rates as low as 1.4% [134]. Band migration can result in acute postoperative stoma obstruction, although this can occur in the absence of migration due to impacted food boluses [135].

Infections of the adjustment port can be an early or late complication. A late adjustment port infection can present years post-operatively, with abdominal pain or port site erythema, caused by band erosion with ascending infection, in up to 1% of cases [136]. This can result in intra-abdominal sepsis requiring removal of the band and high dose intravenous antibiotics. Band erosion is associated with surgical experience, with higher rates in those with less experience [137].

There is a recognition that AGB can have high failure rates in long-term follow-up, although this can likely be remediated by good quality post-operative care [138,139]. The emergence of SG as a procedure with greater weight loss and metabolic effects than AGB, with complication rates comparable or possibly slightly lower than RYGB has led to the consideration of SG before AGB in some cases [139]. However, SG's major Achilles heel is the lack of long-term data with some authors concerned that the 10-year re-operation rate after SG will be similar to that of AGB.

As long-term data accumulate, SG may come to replace AGB in many cases, although for now AGB is likely to remain more popular given the established experience in its use and the lower complication rate in comparison to the other major bariatric modalities [139].

### Nutritional and gastrointestinal complications after bariatric surgery

Deficiencies of iron, vitamin B12, folate, and fat-soluble vitamins can occur after bariatric surgery and are best described in RYGB, BPD and BPD-DS [140]. Vitamin D deficiency can persist despite prescribed replacement in BPD and may tend towards secondary hyperparathyroidism [140]. The risk of nutritional deficiencies depends on postoperative weight loss, the surgical procedure performed and patient compliance with follow up [140,141].

Vomiting is frequent after bariatric surgery but must always be considered to be pathological until proven otherwise after RYGB. An examination and appropriate radiological studies to screen for stricture, stoma stenosis or herniation must be completed. If no pathological cause is found then treatment should be conservative with replacement of fluid and electrolytes [141]. Often, vomiting can be the result of overeating or rapid eating. The patient should be re-educated on eating habits and kept under review.

Diarrhea is reported in up to 40% following bariatric surgery [142]. More than 30% of bariatric surgical recipients report worsening bowel function in the post-operative period and some develop fecal incontinence [143]. The etiology of this is unclear and treatment is based on appropriate dietary modification and anti-diarrheal pharmacotherapy.

There is a variable incidence of the dumping syndrome after bariatric surgery, particularly in RYGB [144]. Dumping syndrome remains a 'waste-basket diagnosis', with the clinical presentation generally considered to include early abdominal pain, diarrhea, nausea, bloating, fatigue, facial flushing, palpitations, hypotension and syncope after high glycemic index meals. These symptoms usually occur within an hour of eating. Similar symptoms that occur two or three hours after a meal include perspiration, palpitations, hunger, tremor, agitation, and syncope [144]. These have been blamed on hypoglycemia and GLP-1, although a definitive etiology remains to be established [145,146].

The treatment of early dumping syndrome is usually straightforward dietary modification, with small regular meals containing protein and carbohydrate with a very low glycemic index. Treatment of the symptoms that occur within two or three hours of a meal also rely on the same dietary modifications, with the added aim of 2 or 3 kg of weight gain which often abolishes the symptoms secondary to the small amount of increased insulin resistance. Pharmacotherapy with acarbose or somatostatin analogues may be needed [147], with transient enteral feeding required in severe cases [144].

There is some overlap in the hypoglycemic syndromes associated with bariatric surgery, with a number of mechanisms likely contributing to each. Obesity-related beta-cell hypertrophy that does not fully regress after weight loss, with improved GLP-1 dynamics and improved peripheral sensitivity, all probably contribute to each syndrome [148]. There can be an exaggerated incretin response in those with hypoglycemic syndromes [145,146]. However, the extent to which the incretin effect is involved can vary by syndrome and even by case [148]. If a post-operative patient presents with dumping syndrome or hypoglycemia that is unresponsive to dietary modification or 3 kg weight gain, or with atypical features such as fasting symptoms, then a full investigation of their insulin dynamics is needed.

### Other complications after bariatric surgery

Other post-operative complications include alopecia, cholelithiasis and hypoglycemia. Postoperative hair loss has been reported in up to 4.5% of bariatric surgical candidates [149]. This is usually mild and non-progressive. Cholelithiasis can occur in up to 2% of individuals in the months after surgery [150]. Ursodeoxycholic acid has been recommended for prevention [140].

## Future directions

As obesity continues to become more prevalent, bariatric surgery will become necessary for greater numbers of people. The current guidelines are aimed at a grade of obesity considered moderate to severe, but evidence is accumulating for the use of bariatric surgery in those with BMI levels of less than 35 kg/m^2 ^[151]. This is particularly the case in those with diabetes [151].

The ongoing randomized controlled trials comparing bariatric surgery and intensive medical glycemic therapy may yield results that will lead to bariatric surgery being used for metabolic benefits in those with diabetes, including those with BMIs of less than 35 kg/m^2 ^[63,64]. This is the continuation of the concept of metabolic surgery; the idea that bariatric procedures should have a primary goal of inducing remission of metabolic diseases, such as diabetes. However, this concept remains controversial, and further data need to be collected to determine the benefits of this approach.

Finally, the goal of individualizing bariatric treatment and predicting response remains challenging. The work on genotyping and predictive models is ongoing but usable models remain elusive. Evidence to date suggests that the factors most predictive of weight loss may be psychological [32]. As genome association data are gathered we may identify genes that can allow us to tailor the bariatric approach to the individual [36,37,152,153]. However, given the expense associated with this technology, its clinical utility at present is low and clinical evaluation will remain the mainstay of pre-operative assessment and procedure selection.

## Conclusions

Bariatric surgery should be considered in individuals with a BMI of greater than 40 kg/m^2 ^and in those with a BMI of more than 35 kg/m^2 ^and obesity related co- morbidities. In future, guidelines may recommend surgery for those with BMIs of less than 35 kg/m^2 ^with diabetes or other metabolic disease.

Not all candidates are suitable to proceed to surgery, and an experienced multi- disciplinary assessment is essential to select the appropriate candidates. The choice of surgical modality should take the individual's goals, surgeon's experience and existing co-morbidities into account. Individualizing care is central to the assessment, and this is determined by clinical evaluation rather than using predictive models, genotyping or biomarkers at present.

Bariatric surgery performed in experienced centers has a low complication rate and leads to long-term weight loss, with associated functional, metabolic and psychological improvements. Bariatric procedures are effective in treating and preventing many obesity related co-morbidities. Long-term follow-up is mandatory to support a safe outcome.

## Abbreviations

AGB: adjustable gastric banding; BMI: body mass index; BPD: biliopancreatic diversion; BPD-DS: biliopancreatic diversion with duodenal switch; GERD: Gastro-esophageal reflux disease; GLP-1: glucagon-like peptide 1; HbA1c: glycosylated hemoglobin; OSA: obstructive sleep apnea syndrome; RYGB: Roux-en-Y gastric bypass; SG: sleeve gastrectomy; T2DM: type 2 diabetes mellitus.

## Competing interests

The authors declare that they have no competing interests.

## Authors' contributions

KJN reviewed the published literature for this review and drafted the manuscript. TO contributed to the content and structure of the article and, in particular, to the sections on surgical technique and complications. CWleR was invited to submit this review, and conceived the design and structure of the article. All authors edited, read and approved the final manuscript.

## Authors' information

KJN is a research fellow in metabolic medicine and has a clinical background in endocrinology and diabetes. His special interests include obesity and the metabolic effects of bariatric surgery, and the effects of the incretin system in obesity and diabetes.

CleR is the Professor of Experimental Pathology at University College Dublin with a special interest in the mechanisms and clinical application of bariatric surgery. His work has focused on how the operations allow long-term weight loss maintenance and metabolic benefit.

TO is a specialist bariatric surgeon who has performed more than 3,000 bariatric procedures. His practice is focused on laparoscopic gastric bypass, sleeve gastrectomy and duodenal switches. He completed his PhD at Sahlgrenska University Hospital in Sweden, and then became the director of the bariatric surgical unit at Carlanderska Hospital in Gothenburg. He is currently leader of a research group in the field of mechanisms and impact of bariatric surgery.

## Pre-publication history

The pre-publication history for this paper can be accessed here:

http://www.biomedcentral.com/1741-7015/11/8/prepub
